# CAPABLE Transitions: A Home Health Agency-Based Intervention to Optimize the SNF-to-Home Transition

**DOI:** 10.1093/geroni/igaa057.3226

**Published:** 2020-12-16

**Authors:** Rachel Missell, Sarah Szanton, Thomas Caprio, Kobi Nathan, Adam Simning

**Affiliations:** 1 University of Rochester, Rochester, New York, United States; 2 Johns Hopkins University, Baltimore, Maryland, United States; 3 St. John Fisher College, Rochester, New York, United States; 4 University of Rochester Medical Center, Rochester, New York, United States

## Abstract

Community Aging in Place-Advancing Better Living for Elders (CAPABLE) consists of an interprofessional team of a registered nurse (RN), occupational therapist (OT), and handyworker that delivers an in-home client-specific package of interventions to optimize function. CAPABLE aims to reduce functional impairment, home hazards, and acute medical services use and is being widely disseminated. To expand CAPABLE to older adults transitioning from the skilled nursing facility (SNF) to home, we developed CAPABLE Transitions, which makes several important modifications to CAPABLE. First, CAPABLE Transitions will be implemented within a Medicare-certified home health agency (CHHA) and delivered to CHHA clients. Second, it will be delivered to CHHA clients with and without dementia. Adding urgency to CAPABLE Transitions’ development, including persons with dementia has the potential to decrease high utilization of services and meet care transition needs. Third, it includes an initial RN care transition visit. Fourth, its services are more intensely delivered at the beginning of the intervention, shortly after SNF discharge. Beginning in the fall of 2020, CAPABLE Transitions will be tested in a feasibility study of 60 older adults discharged from post-acute SNF care to CHHA services in Rochester, NY. We have designed this 3-year feasibility study to consist of yearly recruitment waves that will enable us to iteratively assess and refine the intervention. Following this study, we hope to test CAPABLE Transitions’ effect on improving home time, quality of life, and the use of acute medical services in order to assist older adults in aging in place.

